# Reinforcement learning in dendritic structures

**DOI:** 10.1186/1471-2202-12-S1-P293

**Published:** 2011-07-18

**Authors:** Mathieu Schiess, Robert Urbanczik, Walter Senn

**Affiliations:** 1 Department of Physiology, University of Bern, Bern 3012, Switzerland

## 

The discovery of binary dendritic events such as local NMDA spikes in dendritic subbranches led to the suggestion that dendritic trees could be computationally equivalent to a 2-layer network of point neurons [1], with a single output unit re presented by the soma, and input units represented by the dendritic sub-branches where synapses are clustered[2]. In a such architecture, NMDA spikes transport information from synaptic input into action potentials.

Although this interpretation endows a neuron with a high computational power, it is functionally not clear why nature would have preferred the dendritic solution with a single but complex neuron, as opposed to the network solution with many but simple units. We show that the dendritic solution has a distinguished advantage over the network solution when considering different learning tasks. Its key property is that the dendritic branches receive an immediate feedback from the back-propagation of the action potential and ( more general, of the somatic membrane potential deflections), while in the corresponding network architecture the feedback would require additional backpropagating connections to the input units. Assuming a reinforcement learning scenario we formally derive a learning rule for the synaptic contacts on the individual dendritic trees which depends on the presynaptic activity, the local NMDA spikes, the somatic action potential, and a delayed reinforcement signal. We test the model for two scenarios: the learning of binary classifications and of precise spike timings. We show that the immediate feedback represented by the backpropagating action potential supplies the individual dendritic sub-branches with enough information to efficiently adapt their synapses and to speed up the learning process. For the binary classifications task, we show that the global performance increased with the number of dendritic sub-branches. We show that spacial information can be stores in precise spike and used in a navigation task.

## References

[B1] Poirazi P Brannon T Mel BW Pyramidal Neuron as Two-Layer Neural Network Neuron 2003 37 989 999 10.1016/S0896-6273(03)00149-1 12670427

[B2] Larkum ME Nevian T Sandler M Polsky E Schiller J Synaptic Integration in Tuft Dendrites of Layer 5 Pyramidal Neurons: A New Unifying Principle Science 2009 325 756 760 10.1126/science.1171958 19661433

